# Reconfigurable Soft Robots by Building Blocks

**DOI:** 10.1002/advs.202203217

**Published:** 2022-10-03

**Authors:** Mohamed G. B. Atia, Abdelkhalick Mohammad, Andres Gameros, Dragos Axinte, Iain Wright

**Affiliations:** ^1^ Rolls‐Royce UTC in Manufacturing and On‐Wing Technology The University of Nottingham Nottingham NG8 1BB UK; ^2^ Rolls‐Royce plc Derby DE24 8BJ UK

**Keywords:** bioinspired, building blocks, modularity, reconfigurability, soft robots

## Abstract

Soft robots are of increasing interest as they can cope with challenges that are poorly addressed by conventional rigid‐body robots (e.g., limited flexibility). However, due to their flexible nature, the soft robots can be particularly prone to exploit modular designs for enhancing their reconfigurability, that is, a concept which, to date, has not been explored. Therefore, this paper presents a design of soft building blocks that can be disassembled and reconfigured to build different modular configurations of soft robots such as robotic fingers and continuum robots. First, a numerical model is developed for the constitutive building block allowing to understand their behavior versus design parameters, then a shape optimization algorithm is developed to permit the construction of different types of soft robots based on these soft building blocks. To validate the approach, 2D and 3D case studies of bio‐inspired designs are demonstrated: first, soft fingers are introduced as a case study for grasping complex and delicate objects. Second, an elephant trunk is used for grasping a flower. Third, a walking legged robot. These case studies prove that the proposed modular building approach makes it easier to build and reconfigure different types of soft robots with multiple complex shapes.

## Graphical abstract

This paper presents the design of soft building blocks that can be assembled and reconfigured to build different modular configurations of soft robots such as robotic fingers and continuum robots. To validate this approach, three case studies of bio‐inspired designs are demonstrated (2D soft fingers, legged robot, and a 3D robotic elephant trunk).

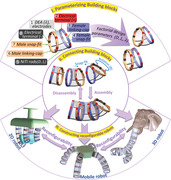

## 1 Introduction

Soft robotics is a relatively recent category where robots consist of compliant structures and are actuated by soft actuation mechanisms. Their construction tackles some of the drawbacks of conventional robots^[^ ^1^, ^2^ ^]^ such as the collision risks during human–robot interaction and lack of flexibility/compliance due to their traditional rigid structures and the reliance on bulky electrical motors. Moreover, soft robots can also be designed to have modularity and reconfigurability abilities which result in improving their flexibility, simplicity of design, easiness in maintenance, and possibility for miniaturizing.

Soft robots can be classified^[^ ^3^, ^4^ ^]^ based on their actuation approaches, namely, i) fluidic elastomeric actuators (FEAs), ii) shape memory alloys (SMAs), and iii) dielectric elastomer actuators (DEAs).

The first group^[^ ^5^, ^6^, ^7^ ^]^ typically uses a vacuum/high pressure for deflating/inflating champers inside the actuators to generate bending movements with a high force to size ratio, for example, 14 N for the size of 50 mm FEA.^[^ ^8^ ^]^ Due to their simplicity in design and actuation, they are widely used in various applications of soft robots such as robotic hands,^[^ ^9^, ^10^, ^11^ ^]^ biomimetic continuum robots (e.g., snake‐like^[^ ^12^, ^13^ ^]^ and elephant trunk robots^[^ ^14^ ^]^ and legged robots.^[^ ^15^, ^16^, ^17^ ^]^ In the case of robotic hands, modular designs have been used to construct multiple configurations of grippers by connecting identical FEA fingers (i.e., modules) in particular positions.^[^ ^18^, ^19^, ^20^ ^]^ Despite the benefits of such modular approaches, studies on the modularity of soft robots are very limited. Further research on FEA focuses on increasing the degree of freedom (DOF) of the soft fingers in 2D, by using modular molds to adjust the lengths of the fingers to generate different behaviors of grasping,^[^ ^21^, ^22^, ^23^, ^24^ ^]^ and in 3D by manufacturing the modules from multi‐materials (e.g., polydimethylsiloxane and ecoflex) where their distribution is optimized to generate diverse bending and twisting movements.^[^ ^25^ ^]^ Nevertheless, the robotic modules are permanently connected in series and, as such, it relies on the independent actuation of each module which limits its ability of disassembly or reconfigurability. As a further development toward increasing the modularity and reconfigurability, identical modules have been proposed to enable the construction of legged, gripper, and continuum robots either, using a LEGO‐like system with bulky interlocking connectors^[^ ^26^ ^]^ or using cylindrical modules with thread connection.^[^ ^27^ ^]^ However, the use of such bulky rigid connectors reduces the flexibility of the system and the initial straight non‐actuated structure limits its applications for complex shapes (e.g., free forms and large curves). FEAs have further limitations due to their nonlinear actuation, air compressibility (making them difficult to be controlled accurately) and the use of ancillary pipes and bulky pumps limit their flexibility. For example, the system developed in ref. [28] has a relatively large module size (45 g‐weight) which limits its extension to applications where the weight is critical.

Soft robots based on SMAs actuation work via the Joule effect,^[^ ^29^, ^30^ ^]^ for example, Nitinol, enable reaching pre‐defined configurations of their structures. Despite their simple design, their applications are limited due to the low strain ratio, slow response time of the material (more than 2 s)^[^ ^29^ ^]^ and the non‐linear response (e.g., elongation) of SMAs. Modular designs have also been presented on SMAs, such as a modular elephant trunk robot^[^ ^31^ ^]^ in which each module consists of several identical and permanently connected segments, another example is a modular robot^[^ ^32^ ^]^ where each module is considered as a whole robotic leg or finger when demonstrated as a walking robot or a gripper, but, these design drawbacks limit their adaptability to the construction of complex configurations, and their reconfigurability.

The third group, the soft robots based on DEAs consists of an elastic backbone frame that is attached to a very thin stretched elastomer membrane (less than 1mm) and sandwiched between two compliant electrodes. A high voltage is applied to the electrodes which generate electrostatic pressure that is converted into strain energy.^[^ ^33^ ^]^ DEA is a promising actuator as it provides the soft robots with; design simplicity, high electromechanical efficiency,^[^ ^34^ ^]^ ease to scale down, low cost, and fast response^[^ ^35^ ^]^ when compared to the previous two approaches. For example, one design^[^ ^36^ ^]^ uses a soft gripper consisting of multiple fingers with PVC material as the backbone frame. By adjusting the frame's width, different performances and curvatures of the finger are obtained, however, the work is done experimentally with no modeling to predict the relationship between the curvatures shape and the design parameters, for example, width, length, and DEA stretching ratios. Some modular designs of DEA have also been presented, such as multi DOF spring rolls^[^ ^37^ ^]^ fabricated to be straight at a non‐actuation state and used to build fish tail fin and legged robots, and manufactured in series to build a snake robot where each spring roll is individually actuated; nevertheless, the relatively long spring roll (90 mm) makes it challenging to achieve complex shapes, the design does not provide a modular method for swapping the spring rolls between different robots configurations, and the straight shape of the robot at non‐actuated state requires too higher voltage to reach its required curved shape which affects the DEA performance and its creep. Another robot design, the elephant trunk,^[^ ^38^ ^]^ was fabricated using one backbone frame that is laser cut with specific patterns to attach a stretched DEA. However, the permanent connections between the modules limit the reconfigurability of the design.

Although all the previously discussed DEA concepts can be applied to flexible frames for the construction of various robots, these works were based on the construction of soft robots with identical sections and constant curvature shapes. More crucially, these research works make a limited emphasis on the prediction of the shape of the robots for given values of the design parameters (e.g., DEA stretching ratios, frame length, and width) and the actuating voltages. Furthermore, the flexible frames of the robots are specific for a robot application, missing modularization concepts to enable shape adaptation for various applications. In the case of complex shapes with variable curvatures, these permanently connected modules require independent actuation and accurate control of each one to follow the required robot shape; resulting in an increased robot complexity with limited flexibility.

Therefore, this paper proposes a reconfigurable modular robotic system actuated by DEA and based on a novel design of soft building blocks that can assemble and construct various types of robots (e.g., robotic hand fingers, continuum robots, legged robots), facilitating the reconfigurability from one assembled robot to another. The novelty resides in the building blocks that can easily snap‐fit not only from a mechanical point of view but also electrically (e.g., electrical terminals) for simultaneous actuation. This technique addresses the drawbacks of the current approaches,^[^ ^37^ ^]^ such as the multitude/bundle of wires when the building blocks are connected in series. A further contribution of this work is that we put a scientific understanding of the relation between the design parameters (e.g., DEA stretching ratio, length, and backbone diameter) and the actuation input (i.e. voltage) with the shape and displacement of a single (and multiple) flexible module(s). Moreover, the building blocks can be rotated with respect to each other to achieve 3D movements of the robot, resulting in a great number of robotic shapes/configurations. A shape optimization algorithm is also presented to support the assembly of the proposed modular soft robots. This algorithm finds the optimal combination of these building blocks to construct specific robot structures based on their required shapes. All these elements of the work allow a conscious design, reducing the trial‐and‐error element from the design process, allowing further development of the concept as a “building blocks toolkit.” Such toolkit is comprised of collections of building blocks of different parameters and shapes, as predicted from the model, for the use in the assembly/construction of the reconfigurable robots. The shape optimization algorithm was demonstrated in a constrained environment on 2D robotic fingers to conform to an object's outer surface, mimicking a human finger. In addition, they are reassembled to form a walking legged robot that can move in 2D plane. To further demonstrate the reconfigurability of the building blocks, they are used to assemble a 3D robotic structure (i.e., mimicking an elephant trunk) that has been demonstrated to grasp and manipulate a delicate object (e.g., flower).

## 2 Results

### 2.1 The Concept of the Flexible Building Blocks

**Figure 1:**
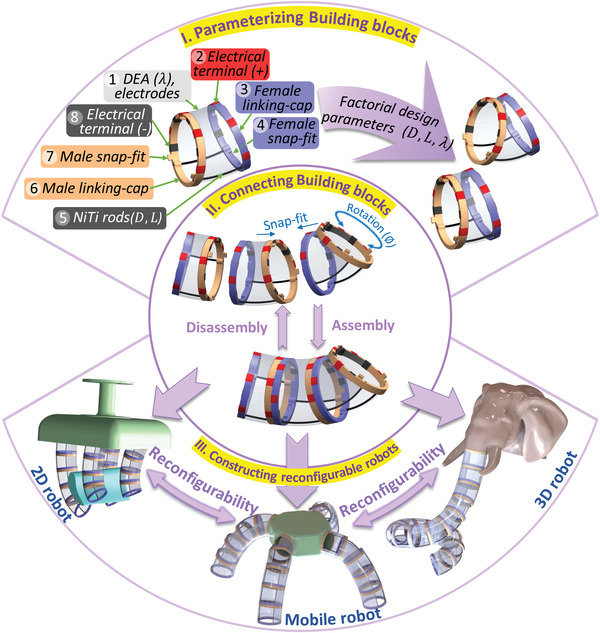
The concept of the reconfigurable modular soft robots by building blocks.

We are proposing a novel concept of reconfigurable soft robots in which the flexible building blocks are the key constitutive elements. The building blocks are soft robotic modules that can be connected in series together to assemble different types and configurations of soft robots like robotic fingers, continuum robots, and legged robots which also can be dissembled and reconfigured into other types and configurations as shown in **Figure** **1**. The novel design of the building block modules consists of four elements: i) a DEA as the sole actuator of the building block (see No. 1 on top of Figure 1); ii) two elastic backbone rods (e.g., NiTi) to generate the spring back force (see No. 5 on top); iii) Two linking‐caps: a male linking‐cap that has three male snap‐fits (Nos. 6 and 7 on top), and a female linking‐cap containing twelve female snap‐fits (Nos. 3 and 4 on top) to enable connecting the building blocks); and iv) electrical terminals to connect all the building blocks with the same applied voltage (see Nos. 2 and 8 on top).

**Figure 2:**
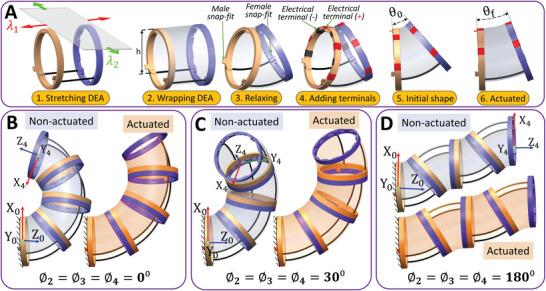
The building block structure. A) A schematic of the manufacturing process (Note: the DEA black electrodes are not showed here for clearer illustrations). The NiTi rods are placed and glued to holes in the linking‐caps while the DEA is stretched planarly (1), the DEA is wrapped around the fixed structure of the linking‐caps with the NiTi rods (2), the building block is released to relax (3), the electrodes are covered to the DEA both sides (assumed transparent for clearer view) and the electrical terminals are added (4), the initial bending angle (5). When a high voltage is applied to the DEA, the bending angle deceases (6). B) The building blocks with zero rotation angles between each other to assemble a constant curvature shape. C) The same building blocks with 30° rotation angles to make a helix shape. D) The same building blocks with 180° rotation angles to make a warm like shape. Note: ϕ_*i*_ is the rotation angle around the local *Z*‐axis of building block *i* in respect to the one before it and $\phi_{1}=0^{\circ }$ for (B–D) cases as the base is fixed.

The initial curvature θ_0_ of the building block is a result of the equilibrium between the stretched DEA before wrapping around the linking‐caps and reaction forces on the two NiTi rods. This initial curvature can be controlled during the manufacturing of the building block to have a certain value by changing the stretching ratios of the DEA (λ), changing the NiTi rod diameters (*D*), or the length of the NiTi rod (*L*) as shown in Figure 1. As proven by finite element method (FEM), these are the key three parameters that affect the curvature, stroke, and stiffness of the building block directly. Their effects can be summarized as follows: i) increasing λ, increases the initial bending angle; ii) increasing the *D*, reduces the initial bending angle and reduces the stroke and the stiffness of the block; iii) increasing *L*, increases the initial bending angle and increases the stroke but reduces the stiffness of the block. While these qualitative dependencies could be intuitive of experienced engineers, precise values are obtained by FEM and summarized in the look‐up table. Other design parameters that could be considered (see details in Section 2.2.1) but kept constant in this research because they could change the design fundamentally, such as the outer diameter of the linking‐caps and the height of the NiTi rod location in linking‐caps (*h*) as shown in **Figure** **2**A.

The actuation of each building block is generated by the DEA wrapped around the linking‐caps; DEA (e.g., ELASTOSILs 2030, VHB 4910) has hyperelastic property that enables its expansion according to an applied high voltage to its sides, hence the actuation of the building block assembly. So, starting from a building block that is manufactured by wrapping a stretched DEA film on the linking‐caps, our concept enables their quick assembly in structures through snap‐fit features as shown in Figure 2A.

The building blocks are actuated simultaneously by the same high voltage through the embedded patterned electrical terminals (next to the mechanical snap‐fits). Each electrical terminal is connected to an outer side of the DEA to ensure the connection of all the outer DEA sides of the assembled building blocks and the same for the inner DEA sides. The process of assembly or disassembly is easily done manually in a few seconds (Movie S1, Supporting Information) through the snap‐fits and the electrical terminals. Whereas the patterned electrical terminals and the patterned 12 female snap‐fits on the linking‐cap of each building block, can be rotated with respect to each other and therefore, the system can be assembled to build a great number of configurations and applications (e.g., 2D, mobile, and 3D robots) as complex as the robotic elephant trunk and it can readily be reconfigured to build a simple 2D finger similar to the robotic fingers. Our building blocks can be assembled to have the desired shape and then actuated together using the electrical terminals to control this shape, thus, our system has simplicity and fewer wiring advantages in constructing the soft robot. Such an approach contrasts with other soft robotic research^[^ ^37^, ^39^ ^]^ where usually independent straight modules are used and actuated individually to reach the required shape. In these approaches, when complex shapes need to be achieved, they might require high strokes/movements on the flexible robot, resulting in high actuating voltages which affect the dielectric eleastomer material creep; jeopardizing its integrity and capability to reach the maximum strain. We believe that a much more elegant way is to take advantage of customizing the design of each building block so that the non‐actuated assembly is similar to the desired shape. The use of bespoke building blocks will allow the complex shape, in actuated form, to be achieved easier.

The building blocks concept for constructing soft robots is not only restricted to circular cross‐sections but it can be extended to include other geometric cross‐sections shapes such as rectangles, flat shapes, ellipses, and triangles to generate similar final shapes to their biological counterparts; depending on the robot application. Nonetheless, in this work, we will adopt a circular cross‐section due to its general applicability.

The reconfigurability can be achieved not only by rotating the building blocks with respect to each other (with angle ϕ) but also, as it will be commented on later, by customizing its curvature (θ) design parameters (e.g., length, NiTi rods diameter, and DEA stretching ratio) to allow multiple building blocks to be manufactured with different shapes and performances. Figure 2B–D shows four identical building blocks assembled in different configurations, where ϕ_*i*_ is the rotation angle between the building block *i* and the block *i* − 1 around the local *Z*‐axis. The configuration in Figure 2B shows the building blocks with zero rotation between them to make a constant 2D curvature shape (experimented in Figure S3I, Supporting Information) and shows the actuation stroke of the system. The second configuration is in Figure 2C and it shows the same building blocks with ϕ = 30° rotations to make a 3D (helix) shape at no actuation and after actuation. The third configuration is in Figure 2D shows rotation between the building blocks of ϕ = 180° that resemble a 2D worm‐like shape to provide a linear motion once actuated with high voltage.

### 2.2 Parameterization of the Building Blocks

As mentioned, the building block is the key constitutive element of the reconfigurable soft robot. In contrast to other reports, we are proposing a modeling approach to understand the initial shape (non‐actuated) and operating voltage stroke (actuated) of the building blocks and linking these properties with their design parameters (e.g., DEA stretching ratio and backbone (NiTi rods) length and diameter). As such, we are taking out the trial‐and‐error approach when designing/constructing this class of robots. In this section, we present a numerical model of a single building block along with 84 different design cases which are applied to a kinematics model to predict the shape of the assembled robot for the different configurations of the building blocks (which are also of different designs).

#### 2.2.1 Step1: Defining the Non‐Actuated and the Actuated States of a Single Building Block

**Figure 3:**
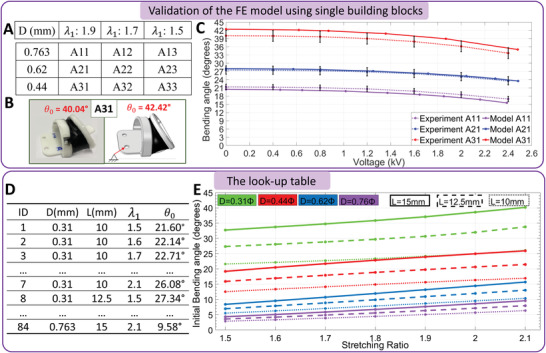
Evaluating the building blocks design parameters. A) The validation matrix that consists of nine building blocks of different parameters. B) An example to show the actual building blocks A31 of the experiment, on left, and the simulation, on right. C) A comparison between the behavior of the experiments and simulation of the first nine building blocks in terms of the bending angle versus the applied high voltage. D) A sample of the look‐up table where ID is the model number, *D* is the diameter of Niti rods, *L* is the active length of the NiTi rods between the two linking‐caps, λ_1_ is the axial stretching ration, and θ_0_ is the initial bending angle. E) The effects of the design parameters to the non‐actuated state of the building blocks from look‐up table.

Based on the design parameters, a finite element (FE) model of a single building block was developed to predict the shape of the non‐actuated (defined by θ_0_) and the actuated (defined by θ) states of the building block. So, we predict a particular state, that is, non‐actuated, of a single block which would enable, later, the customization of an entire robot. The FE model describes the DEA as a hyperelastic material using the widely implemented Ogden model for such material,^[^ ^40^, ^41^ ^]^ the NiTi rods are considered linear elastic elements as their deformation occurs in the elastic region while the linking‐caps are represented by rigid elements. The FE model was validated using a set of nine experiments (example in **Figure** **3**A and details in Figure S8, Supporting Information) and had an average error of 5.8% in angular displacement when compared to the experimental results as shown in Figure 3B,C. The FE model is used to simulate 84 various cases of the building blocks and the results are implemented to develop a “look‐up table;” therefore, significantly reducing the time required for testing.

To reduce the simulation time and to simplify the system, only three key factors are studied as listed below:

i) The distance between the two linking‐caps *L* and has three possible values {10, 12.5, 15} mm. These values represent reasonable values related to the size of the targeted objects to be manipulated (discussed later). Nonetheless, the proposed algorithm and model can be extended to consider more values.
ii) The stretching ratio of the DEA in the axial direction λ_1_ (see Figure 2A) with seven possible values {1.5, 1.6, 1.7, ..., 2.1}. These values were selected as larger stretching ratios will cause collisions between the linking caps and smaller values will generate duplicated results between different NiTi rod diameters. The stretching in the tangential direction λ_2_ is calculated as λ_2_ = 2.5/λ_1_ to have similar thicknesses and operating voltage between the 84 cases.
iii) The NiTi rod diameter *D* and it has four possible values {0.31, 0.44, 0.62, 0.763} mm. Based on the building block behavior from preliminary experiments, these values are selected to develop a wide range of θ_0_ from 3° to 40°.

The parameters kept constant are: i) the outer diameter of the building block (25 mm), selected to facilitate the manipulation of the target objects with sizes ranging from 20 to 50 mm; ii) the vertical shift distance *h* (see Figure 2A) between the circle top and the NiTi rods (8 mm). This value of *h* provides a good balance between the stroke and the size of the DEA; and iii) the length of the linking‐cap (2.5 mm), is selected as it allows embedding the electrical terminals, and the snap‐fit features while providing easy assembly/disassembly.

By using a full factorial experiment, 84 designs of the building blocks are generated and simulated numerically to extract θ_0_ of each structure. Figure 3D presents a sample of the look‐up table (see the full table in Table S1, Supporting Information) while Figure 3E shows the data generated from the look‐up table which presents the relationship between the stretching ratios (λ_1_), the NiTi rods diameters (*D*), and lengths (*L*) to the initial bending angle (θ_0_). It is concluded that the smaller the *D* or the larger the *L*, the larger the θ_0_. For the same ratio (*D*/*L*) of NiTi rod, θ_0_ is increasing linearly with the increase of the λ_1_. The relationship between λ_1_ and θ_0_ is approximately linear for larger values of *D*. It is also noted that different designs can generate the same θ_0_ but with different lengths; for instance, the building blocks with *D* = 0.31 mm and *L* = 10 mm and the building blocks of *D* = 0.44 mm and *L* = 15 mm have similar θ_0_ along with the stretching ratios between 1.9–2.1, (see IDs 5–7 and 40–42 in Table S1, Supporting Information).

When a high voltage (1–2.5 kV) is applied to the building block with an initial bending angle θ_0_, the DEA expands causing relaxation to the backbone elements and, thus, driving the movement of the building block to an angle θ_f_. This behavior is modeled on the FE simulation of building blocks and is compared to the experimental results to show how the model is comparable to the real case. For example, the building block of ID 15 (Table S1, Supporting Information), of parameters: λ_1_ = 1.5, *L* = 15 mm, and *D* = 0.31 mm, developed a θ_0_ = 32.15° at FE simulation versus an experimental angle of 30.70°. However at 2.4 kV, the stroke for the simulation is 5.29° while the corresponding stroke in the experiment is ≈5.5°. This means that for a single building block, there is a good match between the FE model and the experiments, hence, the FE model can be used further in the following design algorithm of the whole soft robot. Note that the generated look‐up table includes only θ_0_ (the non‐actuated state) and only a few IDs are further investigated and simulated with high voltage (the actuated state) due to the computational resources required to generate all the angle/voltage curves. So, we can predict a particular start state, that is, non‐actuated, of a single block which would enable, later, the customization of an entire robot.

#### 2.2.2 Step2: Predicting the Shape of a Multi‐Building Block Assembly (Non‐Actuated/Actuated States)

The non‐actuated and actuated geometries of multiple building blocks can be simplified and extracted from the kinematics model to show how the different building block configurations affect its shape. The kinematics of the reconfigurable soft robot is derived from the continuum robot kinematics^[^ ^42^ ^]^ although the building blocks bending angles and lengths are derived from the look‐up table of the FE model. The robot is first represented by curves with constant curvatures along the centerline and then the robot's full body can be driven by simple transformations. By neglecting any deformations in the linking‐caps and assuming that the bending of the NiTi rods takes constant curvature form, the building block is represented by a straight line followed by a tangent arc and then a tangent straight line to represent the first linking‐cap, the bent NiTi rods and the other linking‐cap, respectively (see Figure 2B).

In the case of a robot consisting of four identical building blocks of ID 15 connected in series, the robot's initial bending angle is 130.92° and 122.80° in model and experiment respectively, but when actuated with 2.4 kV the bending angle becomes 107.4° (experiment) and 100.8° (model) with an average error of ≈6.6%. Therefore, it is concluded that the model has an acceptable error, justifying its use to further predict the shape of a robot of a particular configuration and as a base in the shape optimization algorithm (discussed in the following section).

This algorithm is used to predict only the non‐actuated state of the reconfigurable robot due to computational resources. Such limitation is not a concern as the robot is compliant enough to give the required motions.

### 2.3 Shape Optimization of Reconfigurable Soft Robots

The shape optimization of the reconfigurable robot is studied in the non‐actuated state in two cases; the first is a general case that optimizes the robot shape to be similar to a desired shape. The second case is constrained optimization to avoid the robot colliding with structures (i.e., occurs frequently while grasping an object using robotic fingers).

#### 2.3.1 Unconstrained Optimization: Using a Desired Shape for Input

**Figure 4:**
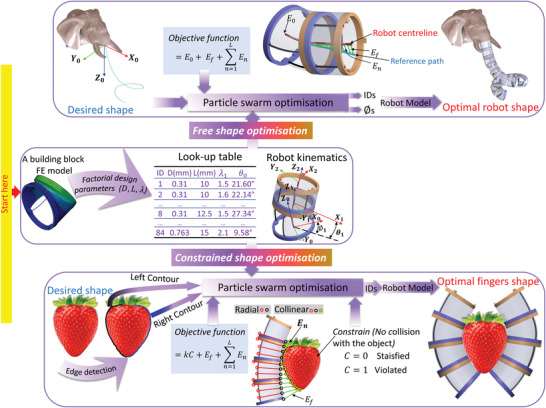
Illustration of the strategy of the shape optimization. The unconstrained shape optimization algorithm of robot configurations is on the top while the constrained shape optimization using grasping strawberry as an example is on the bottom.

The optimization algorithm uses the proposed model to predict the robot shape for specific configurations of building blocks. The algorithm selects, from the look‐up table, the optimal designs of the building blocks that when connected, assemble the shape of the robot to be as close as possible to the required shape; this was achieved by using the particle swarm optimization approach^[^ ^43^ ^]^ as shown in **Figure** **4**.

In the general case of 3D robots to have a desired shape with an optimized shape and no motion restrictions (e.g., collision with other objects), the optimization problem is considered as an unconstrained objective function. The input to the optimization is a desired path of points, defined in the X, Y, and Z coordinates, then the optimization minimizes the distance between this path and the centerline points of the assembled robot model as expressed in Equation (1).

(1) $$\mathrm{Objective}=E_{0}+E_{f}+\sum_{n=1}^{L}E_{n}$$

Where the term *E*_0_ (see Figure 4 on top) is the absolute error between the first point on the robot centerline and the first point on the path, *E*_f_ is the absolute error between the endpoint on the robot centerline and the endpoint on the path, *E*_*n*_ is the absolute error expressed as the distance between a point *n* on the path and a point on the robot centerline which its radial point on the robot periphery is and itself are collinear with the point *n*.

#### 2.3.2 Constrained Optimization: Conforming to an Object Surface Without Collision

This constrained optimization is a special case of the assembly of reconfigurable soft robots where there is a risk of colliding with objects in the environment while following the desired shape, that is to grasp and manipulate an object of a particular shape. In Figure 4, we present a complex case of the modular soft robot optimization that assembles two robotic fingers to conform to a complex object edge in 2D and grasp it: a strawberry.

First of all, the look‐up table of 84 cases is inserted into the algorithm along with a binary image of the object. The number of building blocks in the soft robot is used as an input to the system to allow the user to choose the suitable number of building blocks based on the related application and to reduce the computational resources required. The object image is filtered, and the interior pixels are removed for edge detection of the object shape. Then, the edge is divided into two contours, right and left contours, by removing the extreme pixel on the top and the extreme pixel on the bottom as the grasping is occurring vertically. The image resolution (pixel mm^−1^) is taken into account and the object dimensions are scaled according to this value.

For simplicity reasons, we are giving an example of how to use the optimization algorithm on a 2D shape. As the image of the object is in 2D, the kinematics model of the finger is used in the *X*–*Z* plane only to generate 2D fingers with zero rotation angles (ϕ) between the building blocks. Using the look‐up table and the developed finger kinematics, the shape of the finger is determined and all the points on its periphery so that each two radial points on the periphery are known. Each contour of the object (e.g. right contour) is used in the optimization to generate its optimal robotic finger that conforms to it. For each point on the contour, the corresponding two collinear radial points on the finger that make the three points collinear are determined and their distance is calculated. Additionally, the distance between the endpoint on the finger and the endpoint on the object contour is calculated and added to the accumulative distance between all the points of the object contour and the finger periphery to ensure that the object and the finger are next to each other on the bottom sides for a better conforming for the grasping. Moreover, there is a constrained condition to avoid colliding the finger with the object body which is done by checking the status of all the coordinate points of the finger body inside the object binary image and expressing it as a logical parameter *C* in Equation (2).

(2) $$C=\left\{\begin{array}{cc} 0, & \mathrm{if}\;\mathrm{the}\;\mathrm{no}\;\mathrm{collision}\;\mathrm{condition}\;\mathrm{is}\;\mathrm{satisfied}\\ 1, & \mathrm{if}\;\mathrm{the}\;\mathrm{no}\;\mathrm{collision}\;\mathrm{condition}\;\mathrm{is}\;\mathrm{violated} \end{array}\right.$$

Merging the accumulative distance and the constrained condition, the objective function is expressed in Equation (3)

(3) $$\mathrm{Objective}=kC+E_{f}+\sum_{n=1}^{L}E_{n}$$

Where *E*_f_ (see Figure 4, bottom) is the absolute error between the endpoint on the finger and the endpoint on the object edge calculated as the distance between them, *L* is the number of points on the object edge, *E*_*n*_ is the absolute error expressed as the distance between the point *n* on the object edge and the corresponding two radial points on the finger periphery where the three points are collinear, and *k* is a penalty factor^[^ ^44^ ^]^ with a large value (e.g., in this research it is 10^11^) to convert the constrained objective function to an unconstrained objective function for the swarm optimization to handle.

With the optimization algorithm of building blocks established, practical examples for taking advantage of this new concept are presented in the following.

### 2.4 Reconfigurable 2D Soft Robots: Finger‐Like Configurations

The first case study application is the reconfigurable soft fingers that can generate 2D bending motion. These soft fingers can be assembled together in parallel to form a robotic gripper that can be attached to the end‐effector of a robotic manipulator for further locomotion of the gripper.

#### 2.4.1 Manipulation of Objects with Predefined Complex 2D Shapes

**Figure 5:**
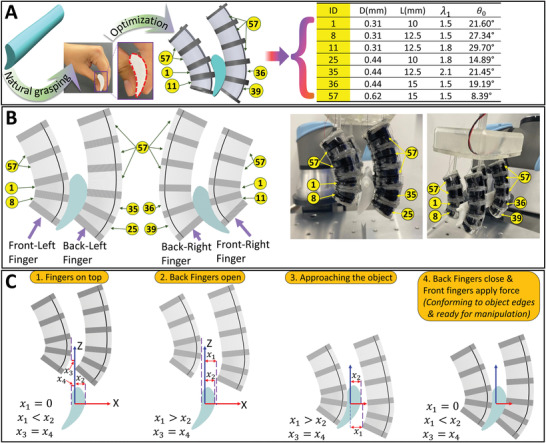
Manipulation of a general complex shape using non‐identical building blocks. A) Grasping of a complex shape using a bio‐inspired technique by the natural grasping of human fingers and the corresponding data from the look‐up table. B) The optimization of its two side views and different views of the experiment. C) The manipulation strategy.

Motivated by the dexterous human fingers which can grasp complex objects by conforming to the shape of the object for power grasping, our reconfigurable soft fingers are designed to imitate this natural grasping technique. This motivation is shown in **Figure** **5**A, which depicts a complex object with different curvatures on both sides, which was picked by the natural way in which the human fingers conform to it. This natural adaptation increases the dexterity and flexibility of the robotic finger, by generating configurations similar to the targeted object surfaces, to safely manipulate the object without dropping it, and widening the range of the manipulated objects (i.e., symmetric and asymmetric objects).

In contrast, most of the current grasping techniques of soft fingers do not geometrically conform to the targeted objects and rely more on the (non‐controlled) deformation of the structure by using excessive values of high voltages. This could result in dropping, applying highly concentrated forces on one point of the object or even its breakage (i.e., some cases of asymmetric and thin objects).

**Figure 6:**
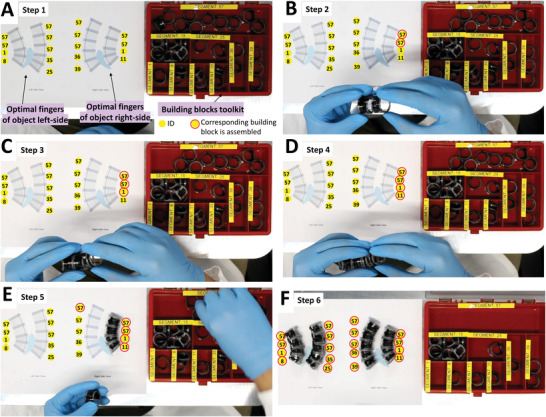
Snapshots of the assembly of non‐identical reconfigurable soft fingers using building blocks toolkit. A) Different building blocks in red box with labels on the right and on the left there is a figure with the desired shapes of soft fingers with their optimized IDs. B) Two building blocks of ID 57 are snap‐fitted and connected together. C) Another building block of ID 1 is connected to them. D) Building block of ID 11 is connected to them to make the soft finger. E) The assembly process is repeated for the remaining three soft fingers. F) The assembled four different soft fingers with non‐identical building blocks.

**Figure 7:**
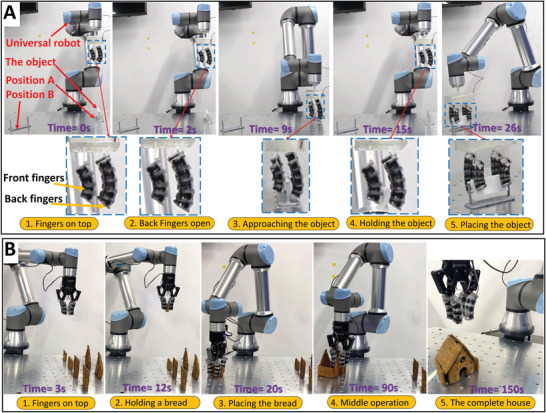
Snapshots of the reconfigurable soft robots in 2D. A) The manipulation of a general complex shape from position A to position B using the optimized reconfigurable robotic fingers. B) The assembly of a gingerbread house using the reconfigurable robotic fingers.

The complex object for this trial consists of a 3D printed asymmetric shape with concave and convex edges, a 15° twist along 50~mm length in the middle and a total length is 90 mm with two different side shapes (see Figure 5B). The left and right‐side views of this asymmetric object are used in the constrained optimization (explained before) to select the optimal building blocks that can be used to assemble four non‐identical fingers for geometrically conforming to the shape. Four building blocks are used in the front fingers (see Figure 5B) while each back finger is comprised of five building blocks, as more actuation stroke is needed on the back to open the gripper. Furthermore, the top two building blocks of the front fingers (i.e., ID of 57 in Figure 5A) are forced to have a high NiTi rod diameter to increase the stiffness of the top part of the finger to be able to hold the weight of the object. For example, in this particular case, building block 57 is used in this constraint since it has *D*~= 0.62 mm and *L*~= 15 mm. The same constraint is applied to the top three building blocks of the back fingers as shown in Figure 5B. **Figure** **6** shows the manual assembly process, using the building blocks toolkit, of the optimized fingers which were used to demonstrate the grasping and manipulation of the complex object (up to 18.3 g) as shown in **Figure** **7**A (Movie S2, Supporting Information). Although in the case of the manipulated object was of a smaller weight (e.g., 9 g), building blocks with reduced stiffness could be used on the top to improve the total stroke (almost the double in case of ID 15 compared to ID 57) on the expenses of reducing the object maximum weight for a successful manipulation. Movie S2, Supporting Information, shows the optimized soft fingers grasping the 3D asymmetric shape with maximum load 18.3 g. Note the clearances necessary for the assembly of the building blocks as well as inertial of the grasped object can affect the stability of the structure during manipulation.

The strategy of manipulation of this complex object (18.3 g) is shown in Figures 5C and 7A where the assembled soft fingers are attached to a gripper base and the end‐effector of the robot manipulator: i) the gripper is attached to a universal robot and it is manipulated to be above the complex object (i.e., *x*_1_ < *x*_2_ in Figure 5C); ii) the back fingers are actuated to open the soft gripper which is then manipulated to reach and surround the object (i.e., *x*_1_ > *x*_2_); iii) the back fingers are deactivated to close the gripper and conform to the object (i.e., *x*_1_ < *x*_2_); iv) the front fingers are actuated to apply blocked force to the object and for firm grasping (i.e., *x*_1_ < *x*_2_); v) the object is manipulated to the required position using the gripper; and vi) the previous steps are done backward to place the object and remove it from the gripper. The applied high voltage during the actuation is a smooth ramp to 2.4~kV and for the deactivation is another smooth ramp to zero voltage (Movie S2, Supporting Information).

#### 2.4.2 Manipulation of Soft Thin Lumpy Objects to Build a 3D Structure

In this experiment, we are further exploring the capabilities of the reconfigurable fingers to manipulate different objects. Here we used a setup of four fingers, each consisting of three identical building blocks, back‐to‐back to grasp the object by only applying force. The setup was used to assemble a gingerbread house (four walls and two roofs). The gingerbreads were homemade to have uneven thicknesses with soft bumpy lumpy surfaces and weights ranging between 35–50 g. A small horizontal flat plastic base is attached to each gingerbread to help it stand due to its uneven base. The manipulation of this kind of object is an ideal case study as it requires delicate handling and a gentle placing of the walls and the roofs. The finger's setup was reconfigured to successfully manipulate the gingerbread in different configurations and positions without dropping it (Movie S4, Supporting Information). In addition, it is used to safely manipulate the four walls and the roofs at their designed positions, despite the difficulty of placing the roofs on top of the walls as shown in Figure 7B.

The strategy of manipulation can be summarized as the following: i) the fingers are attached to the end‐effector of a universal robot manipulator and they have a suitable fixed distance between their top fixed bases larger than the thickness of the object; ii) they approach the object and surround it; iii) once the fingers are actuated, they are relaxing toward the object and apply forces on its surface; iv) the object can be manipulated and moved from one place to another using the robot manipulator; and v) the fingers can be deactivated to place the object.

The gingerbreads resemble “almost” flat objects with small surface deviations. These flat objects are considered the simplest case that can be grasped and the easiest simple case for the shape optimization. The constrained shape optimization was used for selecting the suitable soft fingers while using an extra constrain of using high NiTi rod diameters to increase the fingers stiffness to be stiff enough to grasp these heavy gingerbreads. The optimization generated fingers with identical building blocks of ID 57 in the look‐up table.

The building blocks used in this experiment consisted of softer DEA material (VHB 4910) to improve the performance and stroke. Additionally, the same curvature (θ_0_) of the building block can be generated using different dielectric elastomer materials, as the VHB 4910 material is stretched 7 × 3.5 (axially × tangentially) times while the ELASTOSIL is stretched 1.5 × 1.67 times to manufacture the building block of ID 57, but the building block with VHB had almost double the performance and stroke.

### 2.5 Reconfigurable 3D Soft Robots: Elephant Trunk‐Like Configuration

One of the main advantages of our building blocks is that they can be used to assemble various types of soft robots like the continuum soft robots in 3D. We have built a robotic elephant trunk to prove this concept. It has been used to hold a delicate object (e.g., a flower) and manipulate it between two different locations (Movie S3, Supporting Information). The elephant trunk is assembled using 17 building blocks with different rotation angles (ϕ) around the local *Z*‐axes to construct its complex 3D shape. The 17 building blocks are selected to be decreasing in respect to their backbone flexibility (*D*) gradually from the top (base) to the bottom (end). The first four are building blocks with ID 57 (*D*~= 0.62 mm) to have much stiffness at the base, then it has two following building blocks of ID 36 (*D* = 0.44 mm), finally, the following eleven building blocks are of ID 15 (*D* = 0.31 mm) to have a higher stroke. The robotic elephant trunk was assembled manually using a photo of an elephant holding a flower (≈1.5 g) as a reference, showcasing the building block's ease of assembly and disassembly of complex soft robots.

**Figure 8:**
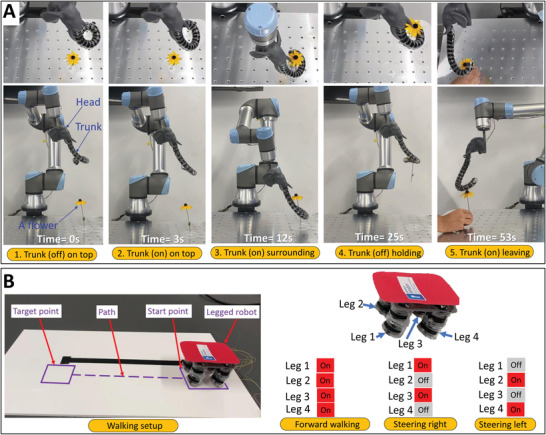
Snapshots of the robotic elephant trunk and the legged robot. A) The robotic elephant trunk manipulated a flower. Top views are on the top while the corresponding front views are on the bottom. B) The legged robot setup and walking gaits.

**Figure** **8** shows snapshots of the manipulation strategy: i) the robotic elephant trunk is on top of the flower in a non‐actuated state (Figure 8A(1)); ii) the robotic trunk is actuated to open (Figure 8A(2)); iii) the robotic trunk is manipulated to approach the flower and surround it (Figure 8A(3)); iv) the robotic trunk is deactivated to close and hold the flower (Figure 8A(4)); and v) the robotic trunk approaches a human hand, actuated to open, give the flower to them (Figure 8A(5)), and then move away from the flower and once it is far enough it can be deactivated to close.

### 2.6 Reconfigurable Soft Mobile Robots: Legged Robot Configuration

To further prove the reconfigurability of the proposed soft robotic system with building blocks, a simple legged robot is assembled and demonstrated. Using the same building blocks used to assemble the robotic fingers in Section 2.4.2, four 1DOF legs (each leg consists of two building blocks) are assembled and attached to a platform to form this simple legged robot as shown in Figure 8B. The maximum speed of the legged robot is around 2.1 mm s^−1^ at 5 Hz. The steering of the legged robot is controlled by supplying H.V. current to the legs or not. The legged robot is demonstrated by following a desired path while steering it (Movie S6, Supporting Information).

## 3 Discussions and Conclusion

This work introduced a novel design of building blocks that can be assembled, disassembled, and reconfigured to construct various robotic types and shapes of varying 2D and 3D complexity. The assembly of the reconfigurable robot relies on snap‐fits to connect the building blocks mechanically in series, and electrical terminals to connect them electrically in parallel to be actuated simultaneously. By changing their design parameters (DEA stretching ratios and backbone length and diameter) various shapes and curvatures of the individual building blocks can be achieved. Moreover, the building blocks can be rotated with respect to each other to form different complex structures which widen the range of the possible robotic shapes that can be constructed using this concept. Another advantage is the lightweight of the building blocks (between 0.9 and 1.1~g for the smallest and largest backbone lengths (*L*~= 10–15 mm) respectively).

Furthermore, a model of the reconfigurable robots is developed based on a combination of a FE model of a single building block and continuum robot's kinematics to show how the design parameters affect the building block curvature and how the various building blocks develop the final shape of the assembled robot. Using this model, we have developed an optimization algorithm to construct reconfigurable soft robots with specific desired shapes in unconstrained and constrained environments.

As a proof of concept, 2D robotic fingers are assembled and reconfigured to suit different objects grasping. Two examples were studied in this research, one was the manipulation of a complex object (19 g) using a combination of the flexural rigidity of the reconfigurable fingers and applying force (inspired by the natural grasping technique of the human hand) where the shape optimization was effective to guide the conforming of the fingers to the complex object surfaces. The second example was done by only applying force for the manipulation of flat thin lumpy objects (50 g) and using them to assemble 3D structures (a gingerbread house). Moreover, a 3D continuum robot of an elephant trunk is developed to grasp a flower using 17 different building blocks that are connected and rotated to each other to generate the complex shape of the robotic elephant trunk simply and easily. Thus, the reconfiguration ability using the building blocks can convert simple robotic fingers to complex‐shaped continuum robots using the same building blocks and vice versa.

Although this research focuses on the setup where the building blocks have electrical terminals to connect all of them electrically to be actuated simultaneously for simplicity of wiring, it is possible to control each building block independently to have more control on the robot shape during actuation. But this requires removing the electrical terminals and connects each DEA of a building block to two thin wires which could lead to increased wiring, short circuits, and sparks.

The building blocks rely on the DEA for actuation such that using different DEA materials significantly affects its actuation performance. For example, the ELASTOSIL material provides a fast‐dynamic response but it generates small stroke of the building blocks as shown on the reconfigurable fingers used in the manipulation of a predefined complex object and the robotic elephant trunk. Although, the building block performance is improved by using a lower stiffness and higher permittivity DEA material for example, VHB 4910 material without making changes to the shape of the building blocks (i.e., the building block with VHB material has a maximum stroke of approximately double of the building block with the ELASTOSIL which increased the load capacity). An example of using the VHB as DEA is the reconfigurable fingers used to assemble the gingerbread house.

Another advantage of the system is its stability over long time. An assembled finger (with VHB 4910 as the DE) can withstand continuous powering of constant H.V. voltages (e.g., 4.5–5 kV) for at least an hour but they will fail after 10–19 min for higher voltages (e.g., 5.5 kV) as shown in Figure S9, Supporting Information. Moreover, twelve building blocks were retested after 7 months and they are still working and generating similar performance (not identical) to the time they were manufactured. The proposed robotic can be further improved by using multi‐layers of DEA. For high load requirements, building blocks of high stiffness (i.e., higher NiTi rod diameter) needs to be selected and used as a constraint in the shape optimization algorithm.

Although the shape optimization method was used successfully in the 2D fingers, it is expandable to construct 3D reconfigurable soft robots (e.g., robotic elephant trunk). However, in this 3D case, it should be taken into consideration that the addition of weight due to multiple building blocks will be addressed in future work. Finally, further future work will also include the expansion of the building block concept for the construction of other biomimetic robots (e.g., snake, octopus, fish, fly) to explore further applications of this modular construction approach.

## 4 Experimental Section

### Fabrication of the Building Block

The fabrication of the building block consisted of the following steps (see details in Section S1, Supporting Information); first, the structure of the building block was 3D printed (Figure S1, Supporting Information). Then, the NiTi rods were inserted and glued into the corresponding holes. After this assembly, the DEA was stretched using a planar stretching mechanism with known stretching ratios in both directions (Figure S2, Supporting Information). After that, a flexible frame was attached to the stretched DEA and it was glued to it on the edges to keep the DEA stretched while bending the frame. Once the glue had cured, the flexible frame was wrapped around the building block structure which had silicone adhesive placed on its linking‐caps. The wrapping process (Figure S3, Supporting Information) was done on supports holding the building block and the flexible frame to keep the stretching in the correct directions. Then, after the adhesive had cured between the DEA and the building block structure, the DEA was cut around the building block to remove any unwanted portions and to remove the building block from the flexible frame. The electrical terminals (i.e., carbon paint) were put on the linking‐caps with a pattern. Each terminal was connected to one side of the DEA. After that, the electrodes (i.e., carbon grease) were painted over the active sides of the DEA in between the linking‐caps. After that, two ultrathin plastic sheets were carefully attached to the linking‐caps above the DEA without contact with its active areas to protect the DEA from contacting any surroundings and the user from touching the DEA and the H.V. as a countermeasure for safe operation.

### DEA Material Characterization

To describe the behavior of the hyperelastic behavior of the DEA material (e.g., ELASTOSIL material), the uniaxial tension and the pure shear tests were performed on samples of 100 µm thickness with dimensions of 20 mm × 5 mm. The experimental setup to characterize the samples consisted of a linear dc motor with a load cell and clamps to fix each end of the DEA sample. A uniaxial test had been performed and shown in Figure S4, Supporting Information, the behavior of the material matched well with the results of ref. [45], while the pure shear test results were similar to the results of ref. [46]. The Ogden model was used to describe the hyperelastic property of the ELASTOSIL material as follows:

i) Uniaxial mode:
(4) $$\lambda_{1}=\lambda_{U},\;\lambda_{2}=\lambda_{3}=\lambda_{U}^{\frac{1}{2}},\;\lambda_{U}=1+\varepsilon_{U}$$
(5) $$T_{U}=\sum_{i=1}^{N}\frac{2\mu_{i}}{\alpha_{i}}\left(\lambda_{U}^{\alpha_{i}-1}-\lambda_{U}^{\frac{-1}{2}\alpha_{i}-1}\right)$$
  where the subscribe U refers to the uniaxial direction, λ_U_ is the stretch, and *T*_U_ is the model stress.
ii) Pure shear mode:
(6) $$\lambda_{1}=\lambda_{S},\;\lambda_{2}=1,\;\lambda_{3}=\lambda_{S}^{-1},\;\lambda_{S}=1+\varepsilon_{S}$$
(7) $$T_{S}=\sum_{i=1}^{N}\frac{2\mu_{i}}{\alpha_{i}}\left(\lambda_{B}^{\alpha_{i}-1}-\lambda_{B}^{-\alpha_{i}-1}\right)$$

where λ_S_ is the stretches in the pure shear, the subscript S refers to the pure shear stretch, and *T*_S_ is the model pure shear stress. Using the particle swarm optimization, the parameters µ_*i*_ and α_*i*_ are optimized to minimize the objective function as shown in Equation (8)

(8) $$\mathrm{Objective}={\left(T_{U.\mathrm{Exp}}-T_{U}\right)}^{2}+{\left(T_{S.\mathrm{Exp}}-T_{S}\right)}^{2}$$

where *T*_U.Exp_ and *T*_S.Exp_ are the experimental uniaxial stress and the pure shear stress respectively, whilst λ_U_ = λ_U.Exp_ and λ_S_ = λ_S.Exp_ and they were the stretch in the uniaxial test and the stretch in the pure shear test, respectively.

The parameters of the third order Ogden model output of the optimization were: µ_1_ = 0.096815~MPa, µ_2_ = 0.14973~MPa, µ_3_ = 0.011493~MPa, α_1_ = 2.8639, α_2_ = 3.23882, and α_3_ = 4.450305.

### FE Model Validation

The FE model (Section S6, Figure S7, and Movie S5, Supporting Information) was validated using nine building blocks of two different parameters: the NiTi rod diameter and the stretching ratio as shown in the validation matrix (Figure S8A, Supporting Information). Each parameter had three different values as follows: i) the stretching ratio in the axial direction (λ_1_ = 1.9, 1.7, and 1.5) while the stretching ratio of the tangential direction (λ_2_ = 2.5/λ_1_) to make the building blocks had similar thickness and operating voltage. A comparison between the experiments and the model in terms of the initial bending angles of the elements A11–A33 is shown in Figure S8B, Supporting Information. The dimensions of the building blocks were scaled up by a factor of 2.5 compared to the reconfigurable modular design to minimize manufacturing errors. Furthermore, the cross‐section shape of the building block was designed as a half‐circle to prove the preliminary concept of the building blocks. VICON measurements were taken where the building blocks were on their side to neglect the gravity effects (Figure S8C, Supporting Information).

The comparison between the behavior of the experiments and the simulations for different applied voltages showed good tracing between them along with the applied voltage (Figure S9A,C,E, Supporting Information). The DEA thicknesses of the different building blocks (Figure S9B,D,F, Supporting Information) measured from the FE model for the applied pressures and used to calculate the corresponding voltage ($V=t\sqrt{P/(\varepsilon_{0}\varepsilon_{r})}$). The average absolute error percentage was 5.8 % and the average absolute error was 1.53° with minimum and maximum absolute errors of 0.11° of 22.17° and 3.08° of 36.34° respectively. While the maximum absolute error percentage was 15.54 % at building block A13 and the maximum absolute error was 3.08° at building block A32.

To increase the accuracy of the measurements and reduce the manufacturing errors, an initial step of calibration was developed where the bending angle was measured after adding the NiTi rods to the linking‐caps and before attaching the stretched DEA. The calibrate angle (zero degrees in an ideal scenario) was measured for each building block in the matrix to compensate for the errors caused by the manufacturing of the fingers and it was in a range of [−2.5°, 2.5°]. The calibrated angle of each building block was added to the bending angles of this building block during actuation to compensate for the manufacturing error of the bending angle versus the voltage by the value of the calibrated angle.

The sources of these errors could be summarized in the following points; first, the manufacturing efficiency was affected by the manually operated stretching device as it had repeatability and accuracy error of max 3%. Second, the optimized hyperelastic parameters of the material caused some errors when compared to the real behavior of the material. Finally, another error source was the estimated elastic behavior of the NiTi rods.

## Conflict of Interest

The authors declare no conflict of interest.

## Author Contributions

M.G.B.A.: Conceptualization, investigation, methodology, experimental work, formal analysis, visualization, and writing—original draft. A.M., A.G., and D.A.: Conceptualization, investigation, research supervision, and writing—review and editing. I.W.: Industrial guidance and funding acquisition.

## Supporting information

Supporting Information

Supplemental Movie 1

Supplemental Movie 2

Supplemental Movie 3

Supplemental Movie 4

Supplemental Movie 5

Supplemental Movie 6

## Data Availability

The data that supports the findings of this study are available in the supplementary material of this article.
