# Supplementary material for: Reconfigurable Soft Robots by Building Blocks
Source: Adv Sci (Weinh). 2022 Oct 3;9(33):2203217. doi: 10.1002/advs.202203217 (PMC9685464; doi:10.1002/advs.202203217)
Supplement: Supplementary file 1 — Supporting Information [file ADVS-9-2203217-s006.pdf]

Supplementary Information

Reconfigurable soft robots by building blocks

Mohamed G. B. Atia, Abdelkhalick Mohammad*, Andres Gameros, Dragos Axinte, Iain Wright

The PDF file includes:

Section S1. The manufacturing process of the building blocks
Section S2. The ELASTOSIL Material characterisation
Section S3. Backbone material characterisation
Section S4. Bending angle measurements
Section S5. Soft robot kinematics
Section S6. The FE model
Figure S1. Preparing the building block structure.
Figure S2. The stretching of the DE.
Figure S3. Gluing the DE to the building block structure.
Figure S4. The ELASTOSIL material characterisation.
Figure S5. The VICON setup.
Figure S6. The kinematics of soft robot.
Figure S7. The FE model.
Figure S8. The validation matrix and setup of the FE model.
Figure S9. The validation of the FE model.
Figure S10. The stability of the DEA under constant H.V.
Figure S11. Blocked force test of initial design
Table S1. The look-up table.

Other Supplementary Information for this manuscript includes:

Movie S1. The assembly and disassembly of the soft robotic fingers.
Movie S2. Manipulation of a complex-shaped object using the robotic fingers.
Movie S3. Manipulation of a flower using the robotic elephant trunk.
Movie S4. Assembling a gingerbread house using robotic fingers with VHB 4910 as the DEA.
Movie S5. Finite element model of a single building block
Movie S6. Walking of the legged robot

Section S1. The manufacturing process of the building blocks:

The manufacturing process of the building blocks involves; first preparing the building blocks structures that each consisting of two 3D printed linking-caps with NiTi rods glued between them (STEP1 and 2) then a silicone adhesive is applied to the outer surfaces of the linking-caps to prepare them to adhere to the stretched DE. The stretched DE is prepared by stretching the DE planarly and then attaching it to a flexible frame from the edges (STEP3), then the flexible frame is wrapped around these linking-caps so that the stretched DE is in contact with the silicone adhesive on the linking-caps. Finally, after the curing time, the DE is cut carefully around the building blocks structures to remove them from the flexible frame, and then the DE is painted with the electrodes (STEP4). The following steps describe the manufacturing process in much detail:

1. ***STEP 1 – 3D printing the linking-caps:***

The linking-caps of the building blocks are prepared by 3D printing using formlabs printer, although other 3D printers can be used as well. The rigid resin or the grey pro resin is used in the formlabs printers. The dimensions of the rigid parts in mm are shown in Figure S1A.

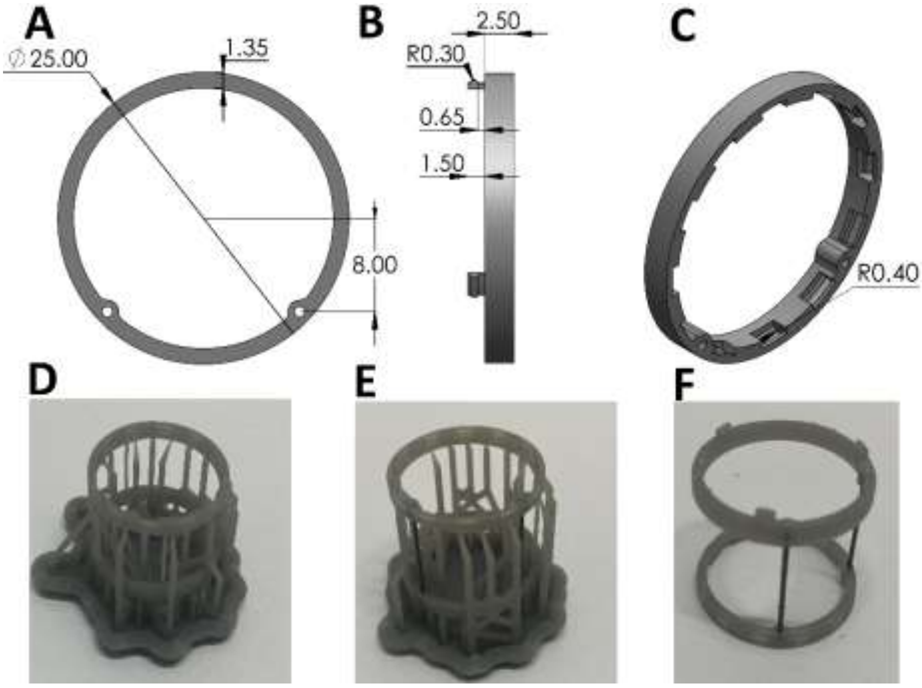

Figure S1. Preparing the building block structure: (A) The outer dimensions of the linking-cap. (B) The male snap-fit. (C) The female snap-fit. (D) The 3D printed structure with supports. (E) The structure with the NiTi rods has been glued to the corresponding holes. (F) The structure after removing the supports.

A snap-fit is designed between the linking-caps so that each building block has a female snap-fit on a linking-cap and a male snap-fit on the other linking cap for assembling and disassembling the soft robots with a clearance of 100μm between the male and female snap-fits as shown in Figure S1B and C.

To ensure that the NiTi rods are in the structure with the desired active length L, the linking-caps are spaced by this distance L during the 3D printing. Figure S1D shows a 3D printed building block with the supports.

STEP 2 – Adding the NiTi rods:

The NiTi rods with specific diameters are inserted and glued, using super glue, to the holes in the linking-caps and left to cure as shown in Figure S1E. Then, after the curing, the supports are removed to obtain the building block structure consisting of the two linking-caps and the two NiTi rods as shown in Figure S1F.

STEP 3 – Stretching the ultrathin silicone sheet:

The stretching of the DE is done using a pantograph mechanism, shown in Figure S2A, to control the stretching in the two planar directions. There are several pre-stretching frames and one stretching frame that are inserted in the pantograph mechanism to restrain its size and the ratio between their sizes gives the stretching ratios of the DE. This stretching frame is with dimensions of 170mm x 120mm and specific slots and it is used to have the size of the stretched DE. Further, the several pre-stretching frames with specific dimensions based on the stretching frame are used as guidance for the pantograph mechanism of its initial pre-stretching position. For example, the pre-stretching frame 2.0 has the dimensions of 85mm x 96 mm is used to build a stretching of 2.0 in the axial direction and 1.25 in the tangential direction. There is another flexible frame with the size of the stretching frame and with heads similar to its slots to attach the stretched DE to its edges from the pantograph mechanism. For more details, the mini-steps of the stretching of the DE of the ratio of 2.0 x 1.25 are listed below:

Step 3.1 – Place the pantograph mechanism tightly around the pre-stretching frame 2.0.

Step 3.2 – The DE is glued to the screws in the pantograph mechanism as shown in Figure S2B.

Step 3.3 – Wait for the silicone adhesive to cure (~20min).

Step 3.4 – The pre-stretching frame is removed from the pantograph mechanism which is then stretched and placed on the stretching frame as shown in Figure S2C.

Step 3.5 – Flip the stretching frame with the pantograph mechanism upside down so that the slots in the stretching frame are accessible from the top as shown in Figure S2D.

Step 3.6 – Insert the flexible frame into the slots from the top after putting silicone adhesive on its edges on the side facing the stretched DE as shown in Figure S2E.

Step 3.7 – The edges of the DE and the flexible frame are pressed gently to make sure that they are both in contact for stronger bond with the silicone adhesive in-between them.

Step 3.8 – Wait for the adhesive to cure (~20min) and then the edges of the DE are cut around the flexible frame to remove it from the pantograph mechanism so that

we have the stretched DE attached to the flexible frame due to the silicone adhesive on its edges as shown in Figure S2F.

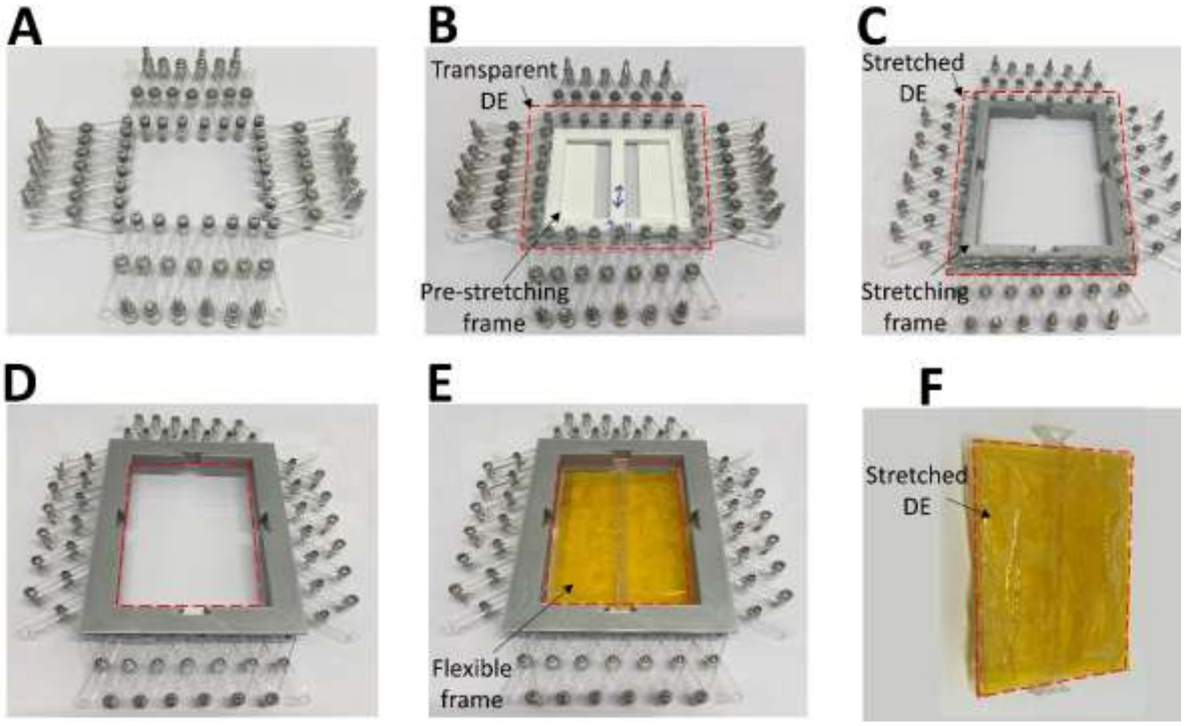

Figure S2. The stretching of the DE: (A) The pantograph mechanism. (B) A pre-stretching frame 2.0 inside the pantograph mechanism and the DE sheet on the top of the pantograph mechanism. (C) The DEA is stretched using the stretching frame. (D) The system is flipped upside down. (E) The flexible frame is placed inside of the frame and glued to the edges of the DE sheet. (F) The flexible frame is removed from the frame.

STEP 4 – Gluing the DE to the building block:

In this step, the flexible frame is wrapped around the building blocks structures where the linking-caps have silicone adhesive applied to their outer surfaces to adhere the stretched DE in the flexible frame to the structure. Then after it cures, the DE is cut around the structures to remove it from the flexible frame and finally the two sides of the DE are painted with the carbon grease.

Step 4.1 – A holder for the building block structure, shown in Figure SA, is used to place and hold building blocks, that are designed to have the same stretching ratio, inside the holder as shown in Figure S3B.

Step 4.2 –A silicone adhesive is placed on the outside surfaces of the linking-caps of the building blocks structures.

Step 4.3 – The flexible frame is inserted in a fixture holder that has similar slots in the flexible frame as shown in Figure S3C and D.

Step 4.4 – The building block holder is inserted in the fixture as shown in Figure S3E.

Step 4.5 – The flexible frame is wrapped around the building blocks holder and clips are used to fix them together as shown in Figure S3F.

Step 4.6 – Wait for curing (~20min),

Step 4.7 – The flexible frame and the building blocks holder are removed from the fixture, then the building blocks holder is removed from the flexible frame leaving the building blocks attached to the DE which is attached to the flexible frame as shown in Figure S3G.

Step 4.8 – The DE is removed carefully from the flexible frame to make identical building blocks as shown in Figure S3H.

Step 4.9 – Finally, the active sides of DE are painted using carbon grease and small thin plastic covers are attached above the linking-caps to cover a part of the DEA and be easy to hold it by the users. Figure S3I shows an example assemble where the four identical building blocks are connected together in series with zero rotational angle between them to make a continuous curvature shape.

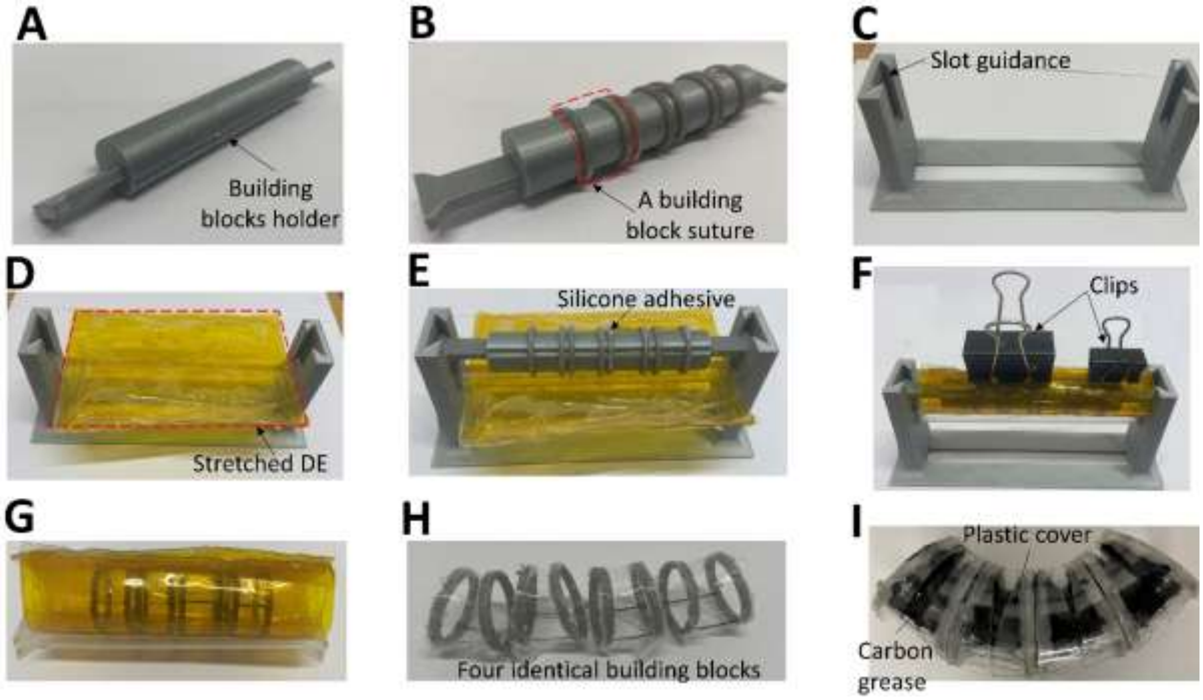

Figure S3. Gluing the DE to the building block structure: (A) The building blocks holder. (B) The building blocks are placed on the holder. (C) A fixture holder. (D) The flexible frame is placed on the fixture. (E) The building blocks holder is placed on the fixture and the silicone adhesive is put on the sides of the linking-caps. (F) The flexible frame is wrapped around the building blocks and two clips are used to fix the flexible frame around the building blocks. (G) The flexible frame and the building blocks holder are removed from the fixture and the building blocks holder. (H) Four identical building blocks after are removed from the flexible frame. (I) The final shape of the building blocks and as an example, they are connected with zero rotational angle between them.

Section S2. The ELASTOSIL Material characterization:

ELASTOSIL 2030 is characterized by uniaxial test and a pure shear test using the experimental setup shown in Figure S4A where a linear DC motor is attached to a load cell which is connected from the other end to a clamp holding the ELASTOSIL sample while the other side of the ELASTOSIL is clamped and fixed. The sample size used for the both tests are 20mm x 5mm. The stress/strain data developed from the two tested are used to optimize the parameters of Ogden model. Figure 4SB and C show a comparison between the experiments and model and the working range of our building blocks is up to strain 1.1 as the axial stretching ratios (λ_1) used in the manufacturing are from 1.5 to 2.1 while the stretching ratios in the tangential direction is 1.19 to 1.67. ($\lambda = \epsilon + 1$)

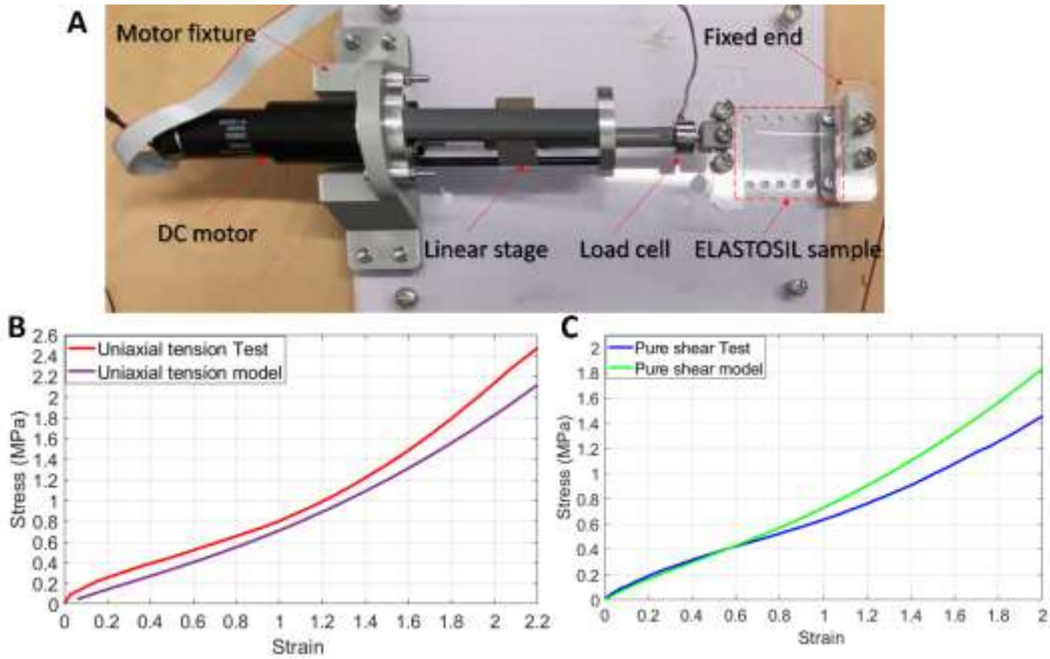

Figure S4. The ELASTOSIL material characterization. (A) The experimental se (B) Comparison between the model and the uniaxial test. (C) Comparison between model and the pure shear test.

Section S3. Backbone material characterisation:

A simple uniaxial tension test is performed on 9 samples of nitinol rods with different diameters (0.763mm, 0.62mm and 0.31mm) to understand the material superelastic behaviour. The elastic region occurs at strains less than 1%. The model assumes that the NiTi behaviour is elastic with an average Young's modulus of 42GPA, since the simulation results show that the deformation of the NiTi is in the elastic region (less than 1%).

Section S4. Bending angle measurements:

The bending angle is measured using the VICON system due to its high accuracy (around 0.1mm) and its flexibility to measure the angles in the three-dimensional axes. The VICON usually consists of four high-speed cameras that have the ability to capture and track the movements of the markers due to their retroreflectivity. Figure S5 shows the VICON setup for validating a building block element from the validation matrix where it is placed horizontally on a fixture holder to neglect the gravity effect and the VICON markers are placed on the back for visibility to the VICON cameras. The VICON uses four markers to track and create a frame. The setup uses two frames each one is attached to a linking-cap where one frame is used as the origin and the VICON tracks and measures the displacement vectors for the other.

But in the case of the reconfigurable modular finger, the other set is placed on a part with snap-fit that is attached to the last building block using the snap-fit. During the measurements, the gravity effect is avoided by placing the building blocks or the finger so that the bending angle is on a plane normal to the gravity vector.

The applied high voltage circuit consists of a XP Power E60 DC to High Voltage DC Converter that is connected to a low voltage power supply. Using the high voltage probe, the output high voltage is measured and calibrated for the system for each input low voltage. The steady-state bending angle is used in the measurements to develop the high voltage vs. the bending angle curves for each step input of the high voltage.

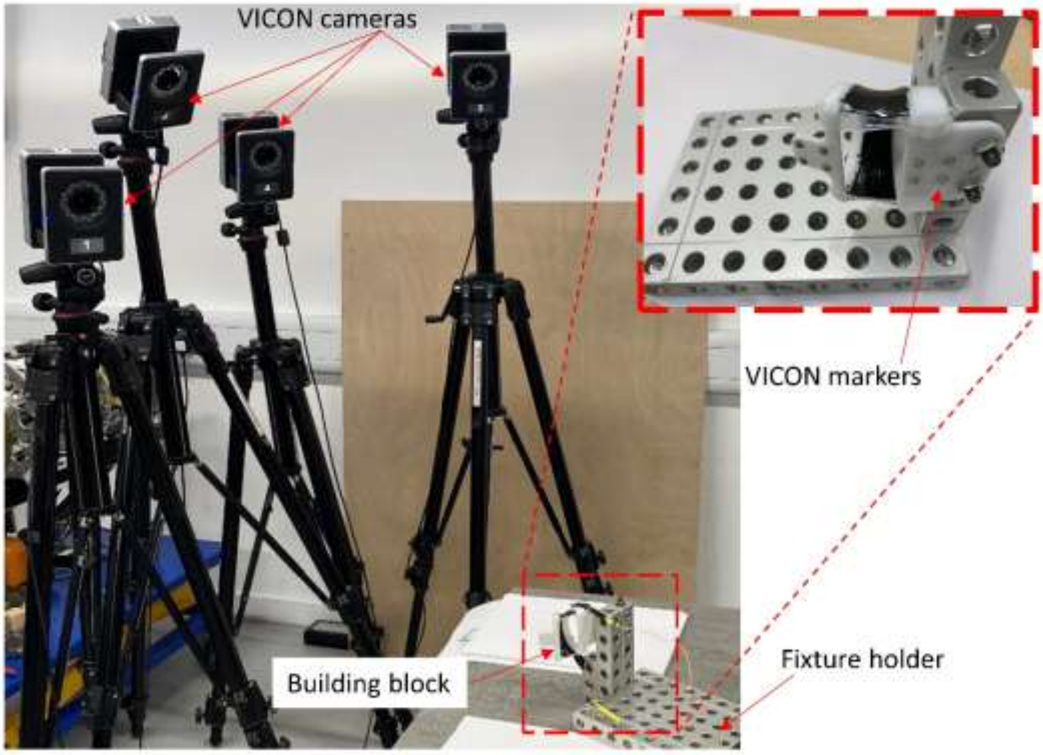

Figure S5. the VICON setup.

Section S5. Soft robot kinematics:

The bending angle of the building block with the order m from the base is θ_m (Fig. S6) and the NiTi rods length between the two linking-caps is L_m which can be extracted from the look-up table, and S_m is the NiTi rods length between the two linking-caps as shown in Equation 1.

$$S_m = \theta_m * \left(\frac{L_m}{\theta_m} - h\right) \tag{1}$$

Where h is the shifted distance between the curved NiTi rod and the centre of the building block (h=8mm in this design) and L_{Rigid} is the length of the linking-cap and is equal to 2.5mm.

First, the linking-caps of building block m are represented by a straight line based on the accumulative bending angle $\boldsymbol{\theta}_{\boldsymbol{m-1}}$ and the accumulative directional angle $\boldsymbol{\phi}_{\boldsymbol{m-1}}$ as shown in Equation 2 and Equation 3:

$$\begin{gathered} \boldsymbol{\theta}_{\boldsymbol{m-1}} = \frac{1}{2}\pi - \sum_{j=0}^{m-1} \theta_j \\ \boldsymbol{\phi}_{\boldsymbol{m-1}} = \sum_{j=0}^{m-1} \phi_j \end{gathered} \tag{2}$$

$$P_{am} = \begin{bmatrix} X_{am} \\ Y_{am} \\ Z_{am} \end{bmatrix} = \begin{bmatrix} L_{Rigid}\cos(\boldsymbol{\theta}_{\boldsymbol{m-1}})\cos(\boldsymbol{\phi}_{\boldsymbol{m-1}}) \\ L_{Rigid}\cos(\boldsymbol{\theta}_{\boldsymbol{m-1}})\sin(\boldsymbol{\phi}_{\boldsymbol{m-1}}) \\ L_{Rigid}\sin(\boldsymbol{\theta}_{\boldsymbol{m-1}}) \end{bmatrix} + \begin{bmatrix} X_{m-1} \\ Y_{m-1} \\ Z_{m-1} \end{bmatrix} \tag{3}$$

Where P_m and P_{am} are coordinates of the first point and endpoint on the first linking-cap of building block m respectively. θ_0 is the rotating angle of the base which is equal to 0.5π when the robot base is vertical. The intermediate points falling on the linking-caps can be represented by spaced points between P_{am} and P_m. As ϕ_m is the rotating angle of the building block around its local z-axis and it is also called the direction angle.

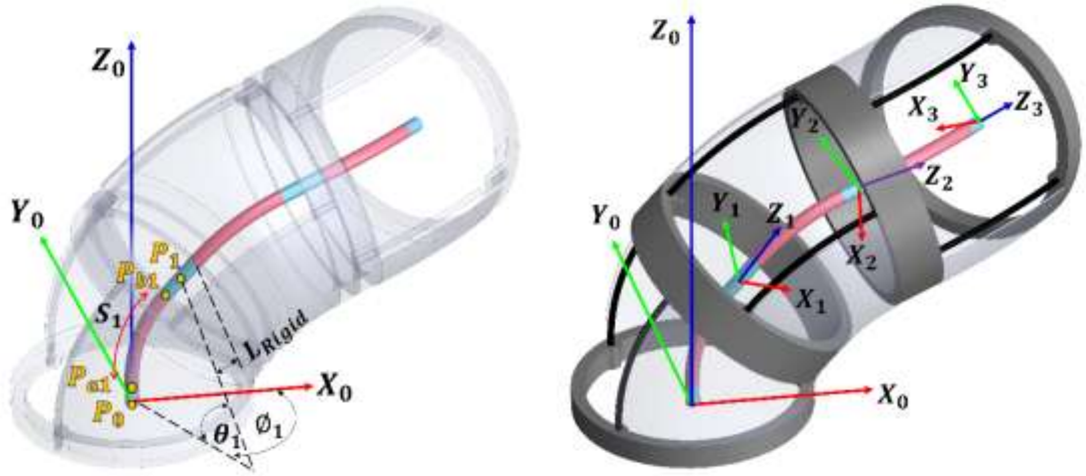

Figure S6. The kinematics of soft robot.

Due to the assumption of constant curvature of the joining NiTi elements of the building block, the general transformation matrix of building block m can be derived in Equation 4:

$$P_{bm} = \begin{bmatrix} X_{bm} \\ Y_{bm} \\ Z_{bm} \end{bmatrix} = R_m \frac{S_m}{\theta_m} \begin{bmatrix} \cos(\phi_m)\,(1-\cos(\theta_m)) \\ \sin(\phi_m)\,(1-\cos(\theta_m)) \\ \sin(\theta_m) \end{bmatrix} + \begin{bmatrix} X_{am} \\ Y_{am} \\ Z_{am} \end{bmatrix} \quad (4)$$

$$R_m = R_{m-1}\ Rot_z(\phi_m - 1)\ Rot_y(\theta_m - 1)$$

Where (X_{bm}, Y_{bm}, Z_{bm}) are the coordinates of the first point on the second linking-cap of building block m and also the endpoint on the bent arc of the same building block.

Similarly, the second linking-cap is represented by a straight line between its first point P_{bm} and the endpoint P_m on building block m as in Equation 5:

$$P_m = \begin{bmatrix} X_m \\ Y_m \\ Z_m \end{bmatrix} = \begin{bmatrix} L_{Rigid}\cos(\boldsymbol{\theta_m})\cos(\boldsymbol{\phi_m}) \\ L_{Rigid}\cos(\boldsymbol{\theta_m})\sin(\boldsymbol{\phi_m}) \\ L_{Rigid}\sin(\boldsymbol{\theta_m}) \end{bmatrix} + \begin{bmatrix} X_{bm} \\ Y_{bm} \\ Z_{bm} \end{bmatrix} \quad (5)$$

Thus, by combining the intermediate points between P_{m-1}, P_{am}, P_{bm} and P_m the soft robot can be represented by a curved centerline across the centre of the building blocks which can be used to describe the complete body of the soft robot.

Section S6. The FE model:

The FE model consists of three quasi-static nonlinear models' analysis in which the data are imported in between them in series (i.e., the model data of the stretched DE is imported as initial state at the second model of gluing and relaxing).

STEP 1 – Stretching model: This model is for stretching the DEA to deform to an arc shape similar to the linking-caps cross-section. The model consists of the DEA which is represented by a 3d deformable element with hyprid formulation mesh and a 3d rigid frame of circular arc to control the shape of the DEA. The DEA is deformed by application of specific distance loads in the directions Y, Z and X (e.g., if the stretching ratio is X in a specific length, the applied displacement load to the perpendicular edge is X-1 times the length) while the contact interface between the DEA and the frame surfaces guides this deformation as shown in Figure S7 A, B.

STEP 2 – Relaxing model: In this model, the DEA is predefined as its initial state is imported from the stretching model and is tie-constrained to the linking-caps of the building block. One of the linking-caps is encastre while the other one is free. Two NiTi rods are inserted as beam elements and tie constrained to their corresponding holes in the linking-caps as shown in Figure S7 C, D.

When the analysis is completed, there is a bending angle (θ) between the two linking-caps that is calculated as the rotation of the rigid part around the perpendicular axis (Y-axis).

STEP 3 – Actuation model: The actuation model is a restart model of the relaxing model where the main difference is that there is an applied pressure on the sides of the DEA that

changes the bending angle of the building block. The bending angle actuation analysis is to evaluate the performance of the building block while one linking-cap is fixed and the other one is free to develop the relation between the developed bending angle versus the applied pressure at no load as shown in Figure S7 E. The corresponding voltage can be calculated to known values of the applied pressure and DEA thickness as shown in Eq. 6.

$$V = t\sqrt{\frac{P}{\epsilon_0 \epsilon_r}} \tag{6}$$

Where ϵ_0 is the permittivity of free space, ϵ_r is the dielectric constant and equal to 2.8 for the of ELASTOSIL 2030 (used in the experiments and validation) and t is the final thickness measured at the end of the simulation at the applied pressure. Due to the non-homogenous thickness, t is calculated as the average of the thicknesses at different locations (at least 5 spots) of the DEA. Therefore, the bending angle is related to the calculated voltage of the corresponding applied pressure. The average measured thickness is shown in Figure S9.

The FE model was developed using Abaqus where the NiTi rods were represented by beam elements with mesh size of 0.5mm. DE was represented by 3D deformable element with hybrid deformation mesh with mesh size of 0.5mm with three layers of local seeds over the thickness. Linking-caps are represented by 3D rigid frames with 1mm mesh size. The FEM used the non-linear analysis due to the expected high deformations of the DE. The mesh size of the DE was 0.5mm with three layers of local seeds over the thickness.

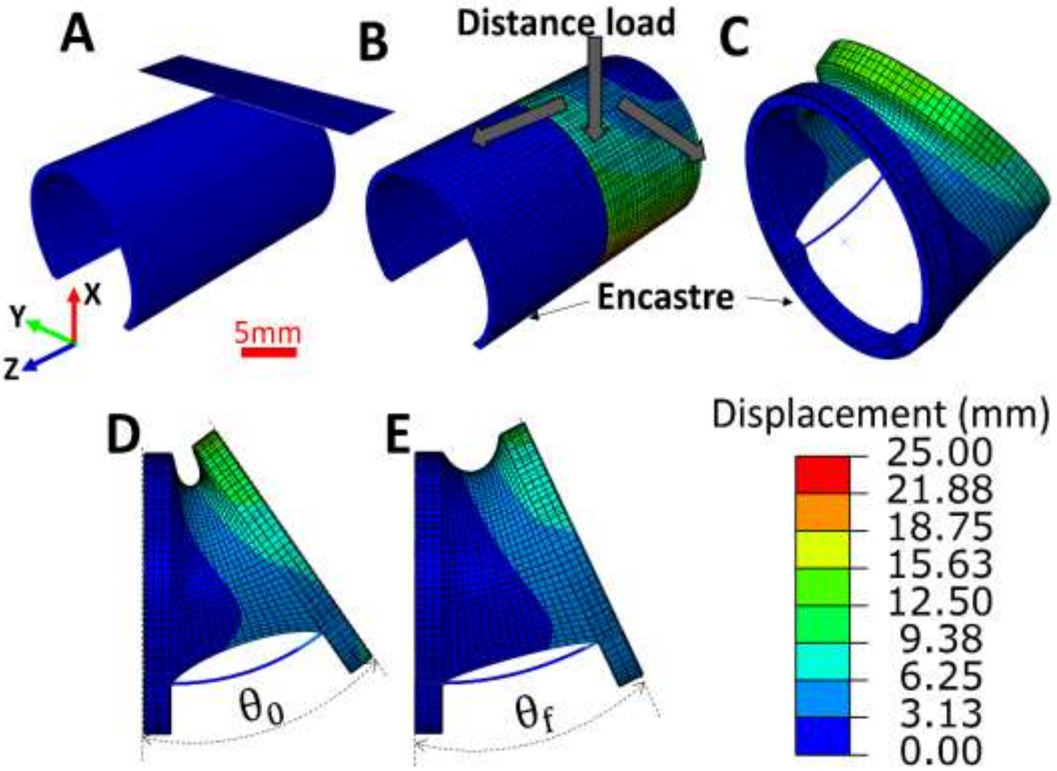

Figure S7. The FE model: (A and B) The stretching of the DEA over a rigid structu (A) Before the stretching. (B) After the stretching. (C) The DEA is translated to the building block with tie constraints between contact of the linking-caps and the DEA, then it is left to relax under no load and as a result, an initial bending angle is develop (D) The bending angle. (E) A hydrostatic pressure is applied according to Equation 6 the upper and bottom surfaces of the DEA causes the bending angle to decrease as an actuation behaviour.

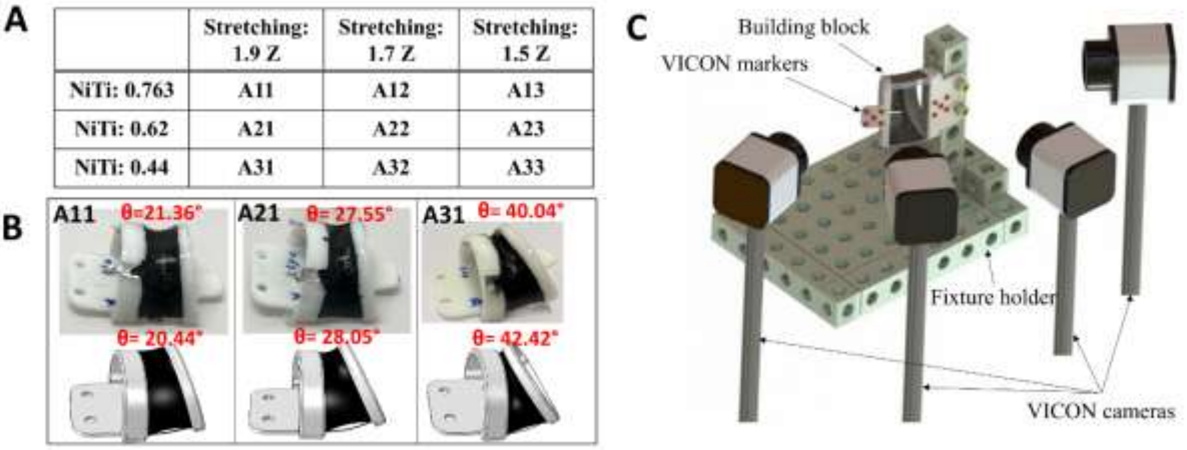

	Stretching: 1.9 Z	Stretching: 1.7 Z	Stretching: 1.5 Z
NiTi: 0.763	A11	A12	A13
NiTi: 0.62	A21	A22	A23
NiTi: 0.44	A31	A32	A33

Figure S8. The validation matrix and setup of the FE model: (A) The validation matrix consists of 9 building blocks of different parameters. (B) An example to show the actual building blocks A-C of the experiments and the simulations. (C) The VICON measurement schematic where the building block is vertical, and the markers are on the bottom.

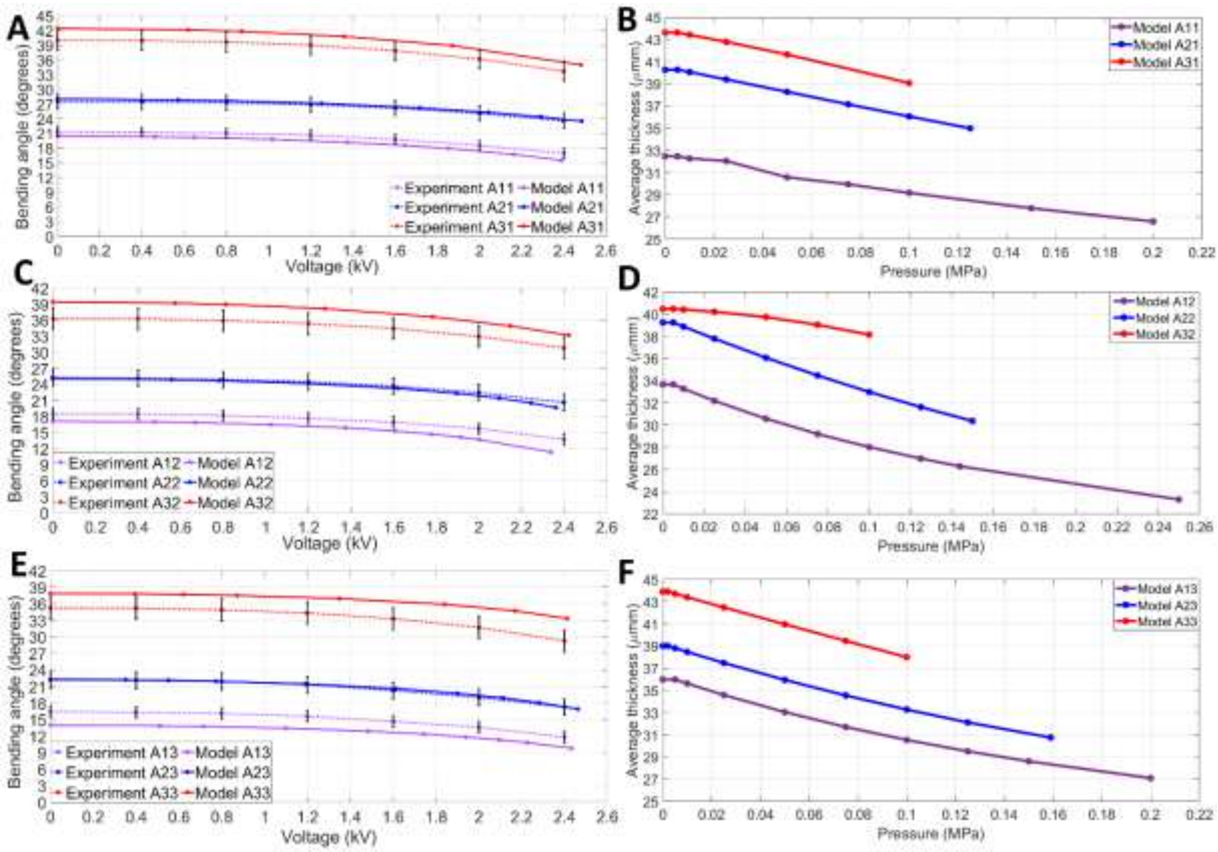

Figure S9. The validation of the FE model: (A, C, E) The comparison between the behaviour of the experiments and simulation of the 9 building blocks in terms of the bending angle versus the applied high voltage. (B, D, F) The measured thickness of the DEA from the FEM compared to the corresponding applied pressure.

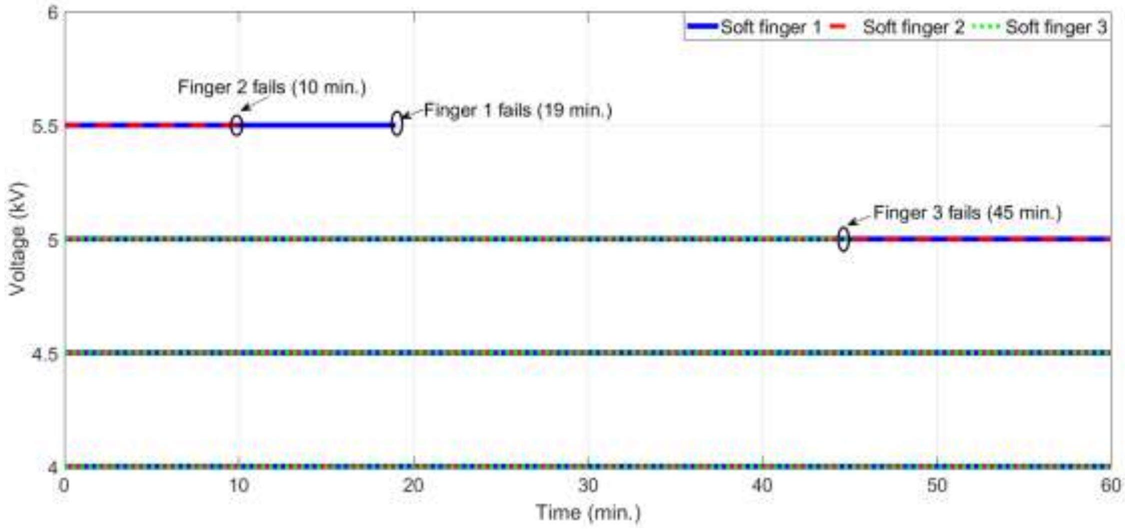

Figure S10. The stability of the DEA under constant H.V.: An indication of the stability of the DEA is shown in the additional Figure S9 below where a constant applied voltage is applied to three almost-identical soft fingers (with VHB 4910 as the DEA) for a period of time to study how stable it before it fails.

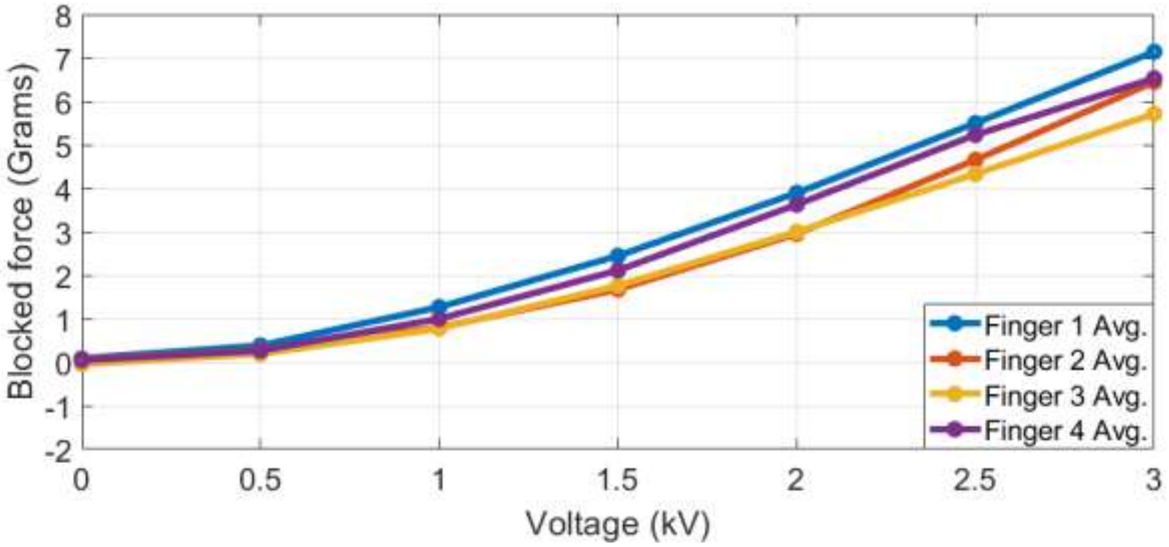

Figure S11. Blocked force test of initial design: This initial design of the soft fingers that have the VHB 4910 as DE and PVC as backbone. To study the effect of the DEA energy only, the blocked force was measured of 4 individual fingers.

Table S1: The look-up table generated from the FE model:

ID	D (mm)	L (mm)	λ_1	Angle(^{0})
1	0.31	10	1.5	21.6
2	0.31	10	1.6	22.14
3	0.31	10	1.7	22.71
4	0.31	10	1.8	23.36
5	0.31	10	1.9	24.16
6	0.31	10	2	24.91
7	0.31	10	2.1	26.08
8	0.31	12.5	1.5	27.34
9	0.31	12.5	1.6	28.07
10	0.31	12.5	1.7	28.84
11	0.31	12.5	1.8	29.7
12	0.31	12.5	1.9	30.68
13	0.31	12.5	2	31.99
14	0.31	12.5	2.1	33.78
15	0.31	15	1.5	32.73
16	0.31	15	1.6	33.74
17	0.31	15	1.7	34.75
18	0.31	15	1.8	35.85
19	0.31	15	1.9	37.08
20	0.31	15	2	38.59
21	0.31	15	2.1	40.16
22	0.44	10	1.5	12.55
23	0.44	10	1.6	13.36
24	0.44	10	1.7	14.14
25	0.44	10	1.8	14.9
26	0.44	10	1.9	15.64
27	0.44	10	2	16.35
28	0.44	10	2.1	16.94
29	0.44	12.5	1.5	15.91
30	0.44	12.5	1.6	16.94
31	0.44	12.5	1.7	17.92
32	0.44	12.5	1.8	18.86
33	0.44	12.5	1.9	19.77
34	0.44	12.5	2	20.64
35	0.44	12.5	2.1	21.45
36	0.44	15	1.5	19.2
37	0.44	15	1.6	20.5
38	0.44	15	1.7	21.71
39	0.44	15	1.8	22.85
40	0.44	15	1.9	23.93
41	0.44	15	2	24.97
42	0.44	15	2.1	25.93
43	0.62	10	1.5	5.5
44	0.62	10	1.6	6.25
45	0.62	10	1.7	7.04
46	0.62	10	1.8	7.85
47	0.62	10	1.9	8.69
48	0.62	10	2	9.52
49	0.62	10	2.1	10.37
50	0.62	12.5	1.5	6.95
51	0.62	12.5	1.6	7.89
52	0.62	12.5	1.7	8.88
53	0.62	12.5	1.8	9.89
54	0.62	12.5	1.9	10.93
55	0.62	12.5	2	11.97
56	0.62	12.5	2.1	13.02
57	0.62	15	1.5	8.39
58	0.62	15	1.6	9.54
59	0.62	15	1.7	10.73
60	0.62	15	1.8	11.95
61	0.62	15	1.9	13.19
62	0.62	15	2	14.44
63	0.62	15	2.1	15.69
64	0.763	10	1.5	2.85
65	0.763	10	1.6	3.34
66	0.763	10	1.7	3.87
67	0.763	10	1.8	4.44
68	0.763	10	1.9	5.05
69	0.763	10	2	5.68
70	0.763	10	2.1	6.34
71	0.763	12.5	1.5	3.6
72	0.763	12.5	1.6	4.2
73	0.763	12.5	1.7	4.87
74	0.763	12.5	1.8	5.58
75	0.763	12.5	1.9	6.34
76	0.763	12.5	2	7.13
77	0.763	12.5	2.1	7.95
78	0.763	15	1.5	4.33
79	0.763	15	1.6	5.07
80	0.763	15	1.7	5.87
81	0.763	15	1.8	6.73
82	0.763	15	1.9	7.64
83	0.763	15	2	8.59
84	0.763	15	2.1	9.58
